# Supplementary material for: New Treatment Strategy Targeting Galectin-1 against Thyroid Cancer
Source: Cells. 2021 May 5;10(5):1112. doi: 10.3390/cells10051112 (PMC8147933; doi:10.3390/cells10051112)
Supplement: Supplementary file 1 [file cells-10-01112-s001.zip › cells-1190884-supplementary.pdf]

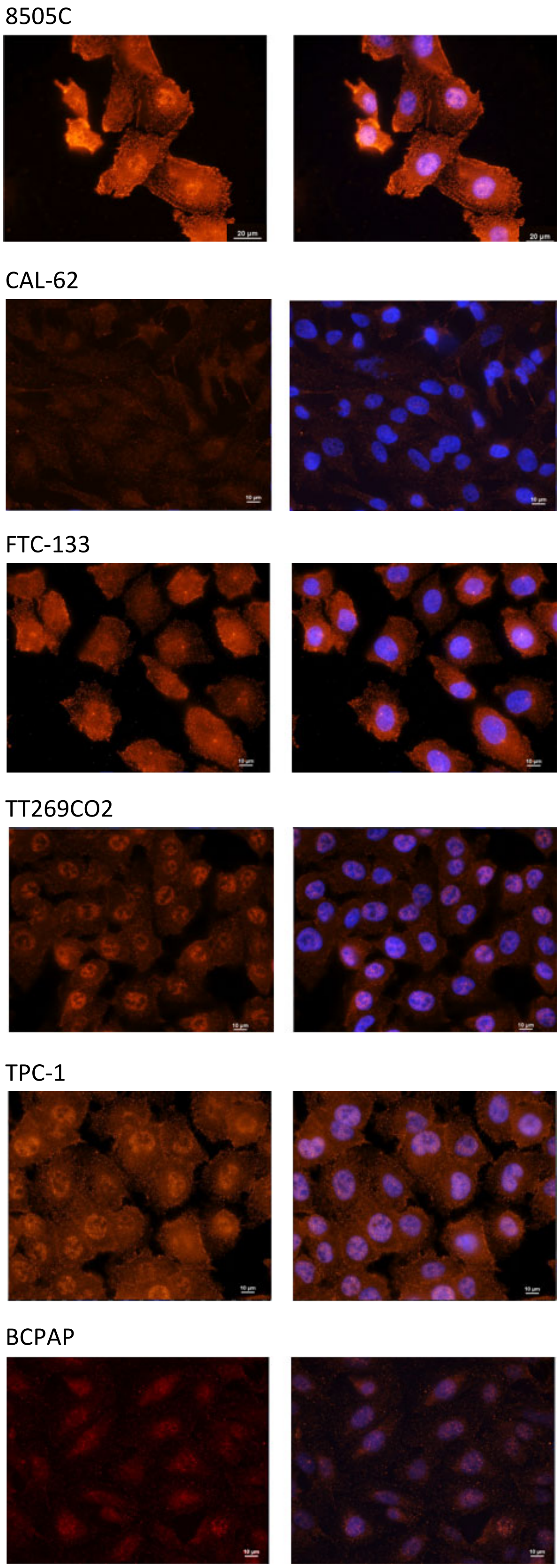

Supplementary Data: Evaluation of Gal-1 expression by immunofluorescence and confocal microscopy. Anti-Gal-1 antibodies were used to detect Gal-1 protein (red staining) in anaplastic cell lines (8505C, CAL-62), follicular thyroid cancer cell lines (FTC-133, TT2609CO2) and papillary thyroid cancer cell lines (TPC-1, BCPAP). DAPI was used to detect nucleus (blue staining).
